# Localization of D-β-Aspartyl Residue-Containing Proteins in Various Tissues

**DOI:** 10.3390/ijms10051999

**Published:** 2009-04-29

**Authors:** Ryota Motoie, Noriko Fujii, Shigeru Tsunoda, Kenji Nagata, Tadashi Shimo-oka, Tadatoshi Kinouchi, Norihiko Fujii, Takeshi Saito, Koji Ono

**Affiliations:** 1 Research Reactor Institute, Kyoto University, Kumatori, Sennan, Osaka 590-0494, Japan; E-Mails: rm.bir.motonari@gmail.com (R.M.); k-nagata@leto.eonet.ne.jp (K.N.); kinouchi@rri.kyoto-u.ac.jp (T.K.); norihiko@rri.kyoto-u.ac.jp (N.F.); ta-saito@rri.kyoto-u.ac.jp (T.S.); onokoji@rri.kyoto-u.ac.jp (K.O.); 2 Graduate School of Science, Osaka Prefecture University, Sakai, Osaka 599-8531, Japan; E-Mail: tsunoda.md@hs.osakafu-u.ac.jp (S.T.);; 3 Life Science Center, Asahi Techno Glass Corp, Funabashi, Chiba 273-0044, Japan; E-Mail: tadashi_shimooka@atgc.co.jp (T.S.)

**Keywords:** d-β-Aspartyl residue, racemization, immunohistochemistry, isomerization, stomach, heart, lung, oxidative stress

## Abstract

Prior to the emergence of life, it is believed that only l-amino acids were selected for formation of protein and that d-amino acids were eliminated on the primitive Earth. Whilst homochirality is essential for life, the occurrence of proteins containing d-β-aspartyl (Asp) residues in various tissues from elderly subjects has been reported recently. Here, we demonstrate the presence of a d-β-Asp-containing protein in the cardiac muscle of heart, blood vessels of the lung, chief cells of the stomach, longitudinal and circular muscle of the stomach, small intestine and large intestine. Since the d-β-Asp residue occurs through a succinimide intermediate, this isomer may potentially be generated in proteins more easily than initially thought. Formation of the d-β-Asp residue in proteins may be related to stress.

## 1. Introduction

Homochirality is essential for life. Before the emergence of life only l-amino acids are thought to have been selected for the formation of proteins, while d-amino acids were eliminated. However, d-aspartic acid (d-Asp) has been detected in various tissues obtained from elderly human subjects, such as tooth [1], bone [2], aorta [3], brain [4,5] and lens [6]. Of all the naturally occurring amino acids, aspartic acid is the most susceptible to racemization. Thus, d-Asp may be formed by racemization in metabolically inactive tissues during the aging process [7]. The *in vivo* racemization of l-Asp in proteins has been investigated over the past two decades. Earlier studies simply measured the d/l ratio of Asp in protein samples, but in subsequent studies the specific d-Asp sites in proteins were reported in lens αA- [8] and αB-crystallin [9], β-amyloid protein of brain [10] and histone of brain [11]. For example, the d isomer to native l isomer ratio (d/l ratio) for Asp-151 and Asp-58 from human αA-crystallin is greater than 1.0, showing that the formation of the d-isomer involves stereoinversion rather than simple racemization. d-Asp formation is also accompanied by isomerization from the natural α-Asp to the biologically rare β-Asp (isoaspartate) residue *via* a succinimide intermediate [8,12]. Therefore, αA-crystallin contains four isomers of Asp: the biologically common residue l-α-Asp, and the unusual residues l-β-Asp, d-α-Asp and d-β-Asp[12]. The formation of these isomers at Asp-151 and Asp-58 of αA-crystallin from human lens begins shortly after birth and continues throughout life until by about 80 years of age so the amount of d-β-Asp exceeds that of normal l-α-Asp [13]. Furthermore, UVB- irradiation of young rat lens induces the racemization of only the Asp-151 residue in αA-crystallin [14]. These results indicate that the configuration of the Asp-151 residue is stereochemically labile, allowing the conversion of l-Asp to d-Asp.

In our previous study, we prepared a highly specific polyclonal antibody against peptide Gly-Leu-d-β-Asp-Ala-Thr-Gly-Leu-d-β-Asp-Ala-Thr-Gly-Leu-d-β-Asp-Ala-Thr (anti-peptide 3R antibody), which corresponds to three repeats of position 149–153 of human αA-crystallin. This antibody can distinguish the configuration of the Asp-residue because it reacts very strongly with the d-β-Asp-containing peptide but not with the l-α-Asp-, l-β-Asp- or d-α-Asp-containing peptides [15]. The antibody also cross-reacted specifically to d-β-Asp-containing protein of lens. Immunohistochemistry demonstrated that the core of the lens from the elderly donors contained d-β-Asp-containing protein, whereas this was not observed in the lens from young subjects. The result was consistent with that of our previous biochemical studies [8]. Therefore, peptide 3R immunoreactive protein can be considered to be d-β-Asp-containing peptide/protein. We have recently detected d-β-Asp-containing protein in sun-damaged skin from elderly donors using the anti-peptide 3R antibody [16]. The abnormal protein was localized in the elastic fiber-like structures of skin samples from elderly donors with actinic elastosis [17]. However, there was no immunoreactivity in sun-exposed skin from young donors [16]. The results clearly indicate that the formation of d-and β-isomers in protein is correlated with both aging and exposure to sunlight. Immunoreactivity of the anti-peptide 3R antibody also depends on the sequence of antigen. Specifically, the antibody recognizes the d-β-isomer of Asp residues in proteins that possess a similar sequence to peptide 3R. Hence, the greater the similarity of the antigen to the sequence of peptide 3R the stronger the anticipated signal intensity. Recently we discovered that many proteins from the NN 1003A cell line, which is derived from rabbit lens, contain d-β–Asp residues [18]. Furthermore, d-β-Asp containing proteins were observed in non-pigmented ciliary epithelial cells, in drusen, in Bruch membrane and in the sclera of human eyes [19]. These results demonstrate that the formation of d-β-Asp residues in proteins is much more widespread than previously thought. Thus, this antibody that specifically recognizes the d-β-isomer of Asp residues is a useful tool for investigating tissue damage caused by aging and stress.

In this article we report the detection of d-β-Asp-containing proteins in various tissues from the whole body of mice using the anti-peptide 3R antibody.

## 2. Results and Discussion

**Figure 1.**
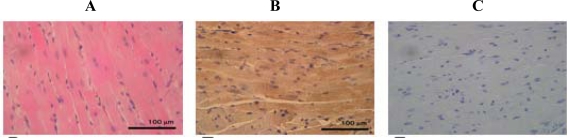
HE staining (A) and immunohistochemical staining (B) of the heart from 6 week-old mice with the anti-d-β-Asp-containing peptide 3R antibody. C: Negative control.

The antibody raised against peptide 3R was used for the immunohistochemical analyses of various mouse tissue samples, including heart, lung, small intestine, large intestine, stomach, kidney, brain, spleen, liver and smooth muscle. d-β-Asp-containing proteins were specifically detected in cardiac muscle, blood vessels of the lung, chief cells of the stomach and longitudinal and circular muscle of the stomach and small and large intestines taken from all age groups of female mice (i.e. 6, 24, 54 and 92 week-old). No immunoreactivity to anti peptide 3R antibody was seen in the liver, spleen, kidney and brain (data not shown). Figures 1A,B shows the Hematoxylin-Eosin (HE) staining, immunohistochemical analysis and the corresponding negative control of heart from a 6 week-old mouse.

Strong immunoreactivity, which stains as brown, was seen in cardiac muscle. This indicates that d-β-Asp-containing protein must be localized in this muscle.

**Figure 2.**
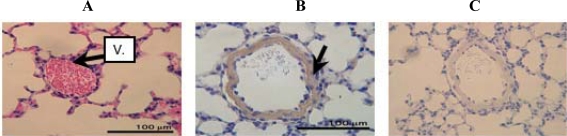
HE staining (A) and immunohistochemical staining (B) of the lung from a 6 week-old mouse with the anti-d-β-Asp-containing peptide 3R antibody. C: Negative control V: blood vessel.

Figures 2A–C shows HE staining and immunohistochemical analysis of the lung of a 6 week-old mouse and the corresponding negative control, respectively. Strong immunoreactivity was observed in the blood vessel (arrow in Figure 2B) of the lung, showing that d-β-Asp-containing protein must be localized in the blood vessel.

**Figure 3.**
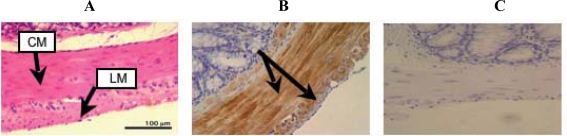
HE staining (A) and immunohistochemical staining (B) of the small intestine of a 92 week-old mouse with the anti-d-β-Asp-containing peptide 3R antibody. C: Negative control. CM: circular muscle. LM: longitudinal muscle.

Figures 3A–C shows HE staining and immunohistochemical analysis of the small intestine of a 92 week-old mouse and the corresponding negative control, respectively.

**Figure 4.**
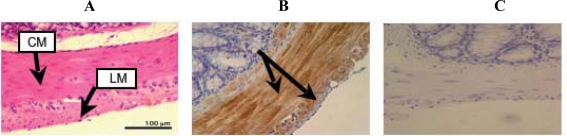
HE staining (A) and immunohistochemical staining (B) of the large intestine of a 54 week-old mouse with the anti-d-β-Asp-containing peptide 3R antibody. C: Negative control. CM: circular muscle. LM: longitudinal muscle.

Figures 4A–C shows HE staining and immunostaining of the large intestine of a 54 week-old mouse and the negative control. Circular muscle and longitudinal muscle in the small and large intestine are strongly stained with this antibody (arrows in Figures 3B and 4B), indicating that the small and large intestine in these organs contain d-β-Asp-containing protein.

**Figure 5.**
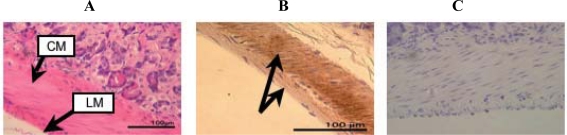
HE staining (A) and immunohistochemical staining (B) of the stomach of a 6 week-old mouse with the anti-d-β-Asp-containing peptide 3R antibody. C: Negative control. CM: circular muscle. LM: longitudinal muscle.

Figures 5A–C represents HE staining and immunohistochemical analysis of stomach tissue from a 6 week-old mouse and the negative control. Strong immunoreactivity to d-β-Asp-containing proteins was seen in the circular and longitudinal muscle (arrows in Figure 5B).

**Figure 6.**
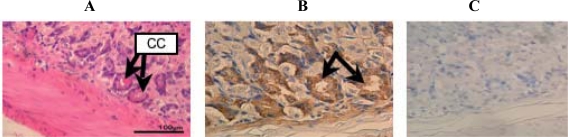
HE staining (A) and immunohistochemical staining (B) of the stomach of a 6 week-old mouse with the anti-d-β-Asp-containing peptide 3R antibody. C: Negative control. CC: chief cells.

The chief cells of stomach were strongly stained blue by HE (Figure 6A). Thus, an antibody reactive against peptide 3R clearly recognizes an antigen in the chief cells of the stomach, indicating that D-β-Asp-containing protein must be localized in this layer (arrows in Figure 6B). The negative control is shown in 6C. Intriguingly, the immunoreactivity observed in the various tissue samples is independent of age. These results are summarized in Table 1.

Table 1 clearly shows that d-β-Asp-containing proteins exist in cardiac muscle, blood vessels of the lung, longitudinal and circular muscle of the stomach, small and large intestine and chief cells of the stomach, regardless of age. In contrast, d-β-Asp-containing proteins were not detected in liver, spleen, kidney and brain. Cardiac muscle, longitudinal muscle and circular muscle are commonly rich in myofibrils, which are covered by connective tissue composed of collagen and elastic fibers. Numerous studies have reported that the Asp residues in collagen are susceptible to racemization [20]. Gineyts *et al.* [21] observed that Asp residues in Type I collagen from the muscle, lung, intestine and kidney were racemized. This is consistent with our present study with the exception of the results from the kidney.

The anti-d-β-Asp-containing peptide 3R antibody clearly recognized antigen in the chief cells of the stomach, indicating that the d-β-Asp-containing protein must be localized in this layer. The area closer to the gastric lumen was much less immunoreactive and the surface of the mucosa was not stained at all.

**Figure 7.**
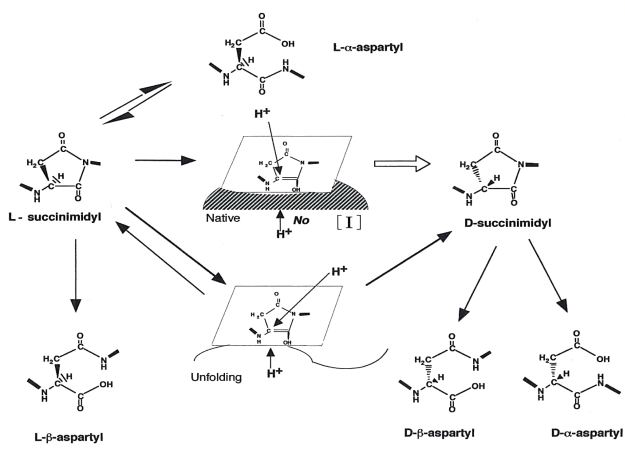
A possible mechanism of isomerization of Asp residues in the protein.

Many reports have shown that the generation of d-β-Asp residues from l-α-Asp or l-α-asparagine (Asn) proceeds readily under physiological conditions *via* a five-membered succinimide ring. As shown in Figure 7, the simultaneous formation of d- and β-residues in proteins could be explained as follows:

Initially, the carbonyl group of the side chain of the l-α-Asp/Asn residue is attacked by the nitrogen of the amino acid residue immediately carboxyl to the Asp/Asn. l-Succinimide, which is formed by intramolecular cyclization, and converted to d-succinimide through an intermediate [I] that has the prochiral alpha-carbon in the plane of the ring. Protonation of the intermediate [I] would occur with equal probability from the upper or lower side of the plane in a peptide or protein (racemization). d- and l-succinimide are hydrolyzed at either side of the two carbonyl groups, yielding both β- and alpha-Asp residues, respectively. The rate of succinimide formation is presumably dependent on the nature of the amino acid neighboring the Asp/Asn residue. When the neighboring residue has a non-bulky side chain, such as glycine, alanine or serine, the formation of succinimide occurs readily because of the absence of steric hindrance [22–24]. Therefore, d-β-Asp formation occurs predominantly in the following protein sequences: Asp/Asn-glycine (Gly), Asp/Asn-alanine (Ala) or Asp/Asn-serine (Ser). Geiger *et al.* showed that the incubation of Asn-Gly-containing hexapeptides at 37°C and pH 7.4 resulted in the formation of 4% d-Asp residues after only one week [22]. It is well known that the chief cells, which are found at the base of the gastric gland, secrete pepsinogen. Chief cells release pepsinogen from granules within their apical cytoplasm *via* exocytosis into the lumen of the gastric gland. In the lumen, the secreted HCl activates pepsinogen to pepsin, which can hydrolyze peptide bonds to initiate protein digestion. Pepsinogen C, has a molecular mass of 42,849 Da and consists of 392 amino acid residues including 14 Asp and 15 Asn (see SWISS-PROT database). Several of these Asp/Asn residues occur in sequences likely to be susceptible to d-β-Asp formation (*e.g*. D73A, D180G, D315S, N224G, N328G, N357A). Therefore, pepsinogen C may be a candidate d-β-Asp-containing protein. Further studies will be required to establish the identity of the d-β-Asp-containing protein and determine the specific site of d-β Asp formation in the protein.

This study clearly demonstrates that d-β-Asp-containing proteins are much more widespread in various tissues than previously thought. The antibody used in this study is very useful for investigating proteins which contains d-β-Asp residues. In this study, the immunoreactivity was located differently in each tissue. In situations where there are a number of proteins which have sequences containing Asp-Gly, Asp-Ser or Asp-Ala, in the tissues, and, if the proteins are also metabolically inactive, the tissues should stain positively. d-β-Asp formation in proteins can cause major changes in structure. Thus, d-Asp formation in a protein can cause large changes in the higher order structure due to the stereochemical changes around the altered residue. Furthermore, β-linkage formation may affect the quaternary structure because the main chain of the protein would be elongated. Therefore, the presence of the isomers may be one of the triggers of abnormal aggregation and may induce the partial unfolding of protein leading to a disease state. In such a state the presence of d-amino acids may be an abnormal protein marker.

## 3. Experimental Section

### 3.1. Animals

Six, 24, 54 and 92 week-old female C3H/HeNJcl mice were purchased from Clea Japan (Osaka, Japan). The mice were housed at 22.0°C in a humidity-controlled room under a 12 hr light-dark cycle (light on at 6:00 a.m.). Food and water was given *ad libitum*.

### 3.2. Materials

The Envision+ system-HRP Labelled Polymer Anti-Rabbit, and the Liquid DAB (3,3’-diaminobenzidine) Substrate Chromogen System was purchased from DAKO Cytomation, Inc. (Osaka, Japan). Buffered neutral formalin solution was obtained from Muto Pure Chemicals Co Ltd. (Tokyo, Japan). Aminosilane-treated glass slides were obtained from Matsunami Glass Ind. (Osaka, Japan). Pronase E from *Streptomyces griseus* was purchased from SERVA Electrophoresis GmbH (Heidelberg, Germany). Mayer’s hematoxylin solution and eosin Y solution were purchased from Wako Pure Chemical Industries, Ltd. (Tokyo, Japan).

### 3.3. Tissue preparation

The 6, 24, 54 and 92 week-old mice were sacrificed by cervical dislocation. The brain, heart, lung, stomach, small intestine, large intestine, kidney, liver and spleen were removed immediately and then fixed in 10% buffered neutral formalin solution for two days at 4 °C. After fixing, the samples were soaked in 70%, 80% and then 90% ethanol for three hours and then twice in 100% ethanol for four hours. The samples were then soaked in toluene and embedded in paraffin. Paraffin sections were cut to a thickness of 4 μm with a microtome and mounted on aminosilane-treated glass slides.

### 3.4. Hematoxylin-Eosin staining

The sections were deparaffinized in xylene and re-hydrated through a graded ethanol series. After washing with distilled water, the sections were soaked in Mayer’s Hematoxylin solution for 18 min at room temperature. The sections were then rinsed several times in distilled water and soaked for two min. Each section was counterstained in eosin Y solution for 20 min, and stained sections were dehydrated through a graded alcohol series.

### 3.5. Antibody against d-β-Asp-containing peptide

The preparation and characterization of an antibody raised against a d-β-Asp-containing peptide has been described elsewhere [15]. The antibody is highly specific against the peptide, Gly-Leu-d-β-Asp-Ala-Thr-Gly-Leu-d-β-Asp-Ala-Thr-Gly-Leu-d-β-Asp-Ala-Thr (designated peptide 3R), which corresponds to three repeats of amino acids 149 – 153 of human alpha A-crystallin optic isomer. The antibody was purified from rabbit serum by affinity chromatography using peptide 3R and bovine alpha A-crystallin as ligands. The antibody clearly recognized the presence of d-β-Asp-containing αA-crystallin in aged human lenses [15]. We also synthesized the peptide IQTGLDATHAER, corresponding to the amino acid sequence 146 – 157 in human αA-crystallin in which Asp residues were either the normal l-α-Asp, or abnormal residues d-α-Asp, l-β-Asp and d-β-Asp. That is, 1) Ile-Gln-Thr-Gly-Leu-l-α-Asp-Ala-Thr-His-Ala-Glu-Arg 2) Ile-Gln-Thr-Gly-Leu-l-β-Asp-Ala-Thr-His-Ala-Glu-Arg 3) Ile-Gln-Thr-Gly-Leu-d-α-Asp-Ala-Thr-His-Ala-Glu-Arg 4) Ile-Gln-Thr-Gly-Leu-d-β-Asp-Ala-Thr-His-Ala-Glu-Arg. The anti peptide 3R antibody clearly distinguished the configuration of the Asp-residue by reacting very strongly with the d-β-Asp-containing peptide but not with the l-α-Asp-, l-β-Asp- or d-α-Asp-containing peptides.

### 3.6. Immunohistochemistry

The sections were deparaffinized in xylene and re-hydrated through a graded ethanol series. Antigens were reactivated by treatment with 0.05% Pronase E at room temperature for 20 min in phosphate buffered saline (PBS). After rinsing in PBS, peroxidase was removed from the cells by addition of 0.3% H_2_O_2_ in 70% methanol. The sample was blocked with 5% skimmed milk in PBS. The sections were then incubated with the anti-d-beta-Asp-containing peptide 3R antibody [15] using a dilution of 1:200 for 24 hours at 4 °C. The sections were washed three times in PBS for five min and incubated in a drop of Envision+ system-HRP labelled polymer anti-Rabbit for 30 min at room temperature. The location of the primary antibody was detected using the Liquid DAB (3,3’-diaminobenzidine) Substrate Chromogen System. The stained sections were dehydrated through a graded alcohol series. To evaluate the specificity of the binding of the primary antibody to the d-β-Asp-containing protein, the sections were incubated with a mixture of the primary antibody and the peptide 3R. The immunoreactions were completely blocked by the incubation (the Figures 1C–6C).

## 4. Conclusions

The present study represents the first reported evidence for the existence of d-β-Asp-containing protein(s) in cardiac muscle, blood vessels of the lung, longitudinal and circular muscle of the stomach, small and large intestine and chief cells of the stomach. Tissues, such as muscle are commonly rich in connective tissues composed of collagen and elastic fibers. The d-amino acids may be formed as a result of racemization in these inert proteins. This study demonstrates the possible widespread presence of d-β-Asp-containing proteins in tissues.

## Figures and Tables

**Table 1.** Summary of Immunohistochemistry Results.

**No**	**age**	**sex**	**brain**	**heart**	**lung**	**stomach**			**small intestine**		**long intestine**		**liver**	**kidney**	**spleen**

				Cardiac muscle	Vessel	Chief cell	Long. muscle	Circ. muscle	Long. muscle	Circ. muscle	Long. muscle	Circ. muscle			
1	6	F	−	**+**	**+**	**+**	**+**	**+**	**+**	**+**	**+**	**+**	−	−	−
2	6	F	−	**+**	**+**	**+**	**+**	**+**	**+**	**+**	**+**	**+**	−	−	−
3	6	F	−	**+**	**+**	**+**	**+**	**+**	**+**	**+**	**+**	**+**	−	−	−
4	24	F	−	**+**	**+**	**+**	**+**	**+**	**+**	**+**	**+**	**+**	−	−	−
5	24	F	−	**+**	**+**	**+**	**+**	**+**	**+**	**+**	**+**	**+**	−	−	−
6	54	F	−	**+**	**+**	**+**	**+**	**+**	**+**	**+**	**+**	**+**	−	−	−
7	54	F	−	**+**	**+**	**+**	**+**	**+**	**+**	**+**	**+**	**+**	−	−	−
8	92	F	−	**+**	**+**	**+**	**+**	**+**	**+**	**+**	**+**	**+**	−	−	−
9	92	F	−	**+**	**+**	**+**	**+**	**+**	**+**	**+**	**+**	**+**	−	−	−
